# Comparison Between Folic Acid and gH625 Peptide-Based Functionalization of Fe_3_O_4_ Magnetic Nanoparticles for Enhanced Cell Internalization

**DOI:** 10.1186/s11671-018-2459-8

**Published:** 2018-02-07

**Authors:** C. Tudisco, M. T. Cambria, A. E. Giuffrida, F. Sinatra, C. D. Anfuso, G. Lupo, N. Caporarello, A. Falanga, S. Galdiero, V. Oliveri, C. Satriano, G. G. Condorelli

**Affiliations:** 1 Dipartimento di Scienze Chimiche, Università di Catania, 95125 Catania, Italy; 2 INSTM UdR di Catania, 95125 Catania, Italy; 3 Dipartimento di Scienze Biomediche e Biotecnologiche, Università di Catania, 95100 Catania, Italy; 4 Dipartimento di Farmacia, Università di Napoli “Federico II”, 80134 Napoli, Italy

## Abstract

A versatile synthetic route based on magnetic Fe_3_O_4_ nanoparticle (MNP) prefunctionalization with a phosphonic acid monolayer has been used to covalently bind the gH625 peptide on the nanoparticle surface. gH625 is a membranotropic peptide capable of easily crossing the membranes of various cells including the typical human blood-brain barrier components. A similar synthetic route was used to prepare another class of MNPs having a functional coating based on PEG, rhodamine, and folic acid, a well-known target molecule, to compare the performance of the two cell-penetrating systems (i.e., gH625 and folic acid). Our results demonstrate that the uptake of gH625-decorated MNPs in immortalized human brain microvascular endothelial cells after 24 h is more evident compared to folic acid-functionalized MNPs as evidenced by confocal laser scanning microscopy. On the other hand, both functionalized systems proved capable of being internalized in a brain tumor cell line (i.e., glioblastoma A-172). These findings indicate that the functionalization of MNPs with gH625 improves their endothelial cell internalization, suggesting a viable strategy in designing functional nanostructures capable of first crossing the BBB and, then, of reaching specific tumor brain cells.

## Background

The blood-brain barrier (BBB) is a dynamic interface between the blood and the brain which plays a crucial role in the maintenance of the central nervous system (CNS) homeostasis. The main components of the BBB are the endothelial cells (i.e., astrocytes and pericytes), which form a continuous sheet covering the inner surface of the brain’s microvessels. They are interconnected by tight junctions, which are critical to both the control of BBB vascular permeability [1] and to the protection of the brain from various circulating toxins and other harmful molecules [2, 3]. Neurons and microglia, which are other neurovascular unit components, play a different role in the BBB [4, 5].

Due to the endothelial cell continuous sheet, 98% of small-molecule drugs and 100% of large-molecule drugs do not pass through the BBB, thus failing to deliver the drugs to the brain tissue [6]. Therefore, in order to develop efficient delivery systems for the treatment of many brain diseases, it is crucial to investigate the carriers’ ability of getting through the BBB [7].

In the last decade, nanoparticle-based systems have been widely studied as effective therapeutic agents for cancer targeting and therapy. In this context, organic functionalized magnetic iron nanoparticles (MNPs) have drawn a lot of interest since they combine the versatility of the surface functionalization with the magnetic properties and the non-toxic biocompatible nature of their core. MNPs are widely adopted for theranostic applications (diagnostic and therapeutic) such as protein and cell sorting and manipulation [8, 9], cell labeling [10, 11], magnetically controlled drug delivery [12, 13], magnetic resonance imaging (MRI) [14, 15], and hyperthermia [16–19]. To be efficiently used for biomedical applications, MNPs should be capable of crossing cell membranes and, in particular, of passing through the various biological barriers. The modification of the MNPs surface with functional molecules has often been used to improve their cell internalization, but in many cases, the crossing of the BBB remains a problem.

In this context, the use of cell-penetrating peptides (CPPs), a group of relatively short peptides (5–40 amino acids) deriving either from natural sources or from synthetically designed constructs, which can easily cross the membrane bilayer, can be seen as a promising approach. Among the numerous available CPPs, the 20-residue peptide gH625, derived from the glycoprotein H (gH) of the Herpes simplex virus 1, has recently been developed and used to cross the BBB to enhance the uptake of a variety of cargoes [20, 21] into the cytosol.

In the present study, MNPs have been functionalized with two different classes of targeting coatings based both on a coupling layer of 3-aminopropylphosphonic acid (NH_2_-PA). The two coatings were obtained by connecting to the NH_2_-PA-modified MNPs (NH_2_@MNPs) either the gH625 (gH625@MNPs) cell penetrating peptide or PEG and folic acid (PEG,FA@MNPs) molecules. In particular, the introduction of folic acid (FA) into the PEGylated nanoparticles aims at improving both the cellular uptake in tumor cells through FA receptor-mediated internalization [22] and the biocompatibility of the overall nanosystem [23]. The effects of the two surface coatings on MNP intracellular uptake into primary microvascular endothelial cells from human brain (HBMECs) have been evaluated by confocal laser scanning microscopy. To this end, luminescent probes were incorporated into both systems. In particular, gH625 was labeled with the 4-chloro-7-nitrobenz-2-oxa-1,3-diazole (NBD) probe, whilst a carboxy-*X*-rhodamine (rhod) probe was added to the shell of PEG,FA@MNPs.

The study of intracellular uptake of gH625-modified MNPs in endothelial cells and their comparison with FA-modified MNPs, to the best of our knowledge, have never been reported before.

Besides the capability of crossing the BBB, targeting brain tumor cells is another important requirement for brain tumor therapeutic agents. Hence, the cellular internalization of the two differently coated MNPs in one of the most common human malignant glioma, the A-172 glioblastoma, was also evaluated.

Note that though gH625-functionalized iron nanoparticles have recently been prepared by Perillo et al. [24], in the present work, we report a different synthetic strategy based on a highly versatile phosphonic acid platform. In particular, phosphonic acid chemistry allows the formation of strong bonds between MNPs and phosphonic monolayers whose stability is comparable to that of silanized MNP. Moreover, since P-O-P self-condensation is negligible, the use of phosphonic linkers overcomes the oligomer formation drawbacks which are often encountered when using silanes [25].

## Methods

### Materials

All the reagents used for MNP synthesis and functionalization, FeCl_2_·4H_2_O, FeCl_3_·6H_2_O, 3-aminopropylphosphonic acid, methoxypolyethylene glycol acetic acid *N*-succinimidyl ester (PEG-NHS) with molecular weight 5000 Da, folic acid, *N*-hydroxysuccinimide (NHS), and carboxy-*X*-rhodamine *N*-succinimidyl ester (Rhod-NHS) were purchased from Sigma-Aldrich and used without further purification. The 9-fluorenylmethoxycarbonyl (Fmoc)-protected amino acids used for the peptide synthesis were purchased by Romil Del Chimica, Chemicals, and were used without further purification. The water was of Milli-Q grade (18.2 MO cm) and was filtered through a 0.22-μm filter.

### Synthesis of MNPs

Bare iron oxide MNPs were synthesized by the alkaline co-precipitation of Fe^3+^ and Fe^2+^, according to the protocol described in the literature [26]. Briefly, FeCl_2_·4H_2_O and FeCl_3_·6H_2_O (molar ratio 1:2) were dissolved in water (50 ml) under an N_2_ atmosphere with vigorous stirring. NH_3_ 25% in H_2_O (5 ml) was added to the solution at 80 °C, and the reaction was continued for 30 min. The resulting suspension was cooled to room temperature and washed with ultrapure water. The obtained bare magnetic nanoparticles (bare MNPs) were isolated from the solvent by magnetic decantation.

### Synthesis of gH625

The peptide was prepared as previously reported [27] adopting the standard solid phase 9-fluorenylmethoxy carbonyl (Fmoc) method. Briefly, 50 μmol of the peptide was synthesized on a Wang resin (0.75 mmol/g) by consecutive cycles of deprotection and coupling. The resin-bound peptide was then reacted with 4-chloro-7-nitrobenzofurazan (NBD-Cl); the reaction was carried out overnight in the presence of DIPEA. The peptide was cleaved from the resin and deprotected by treatment with a mixture of trifluoroacetic acid (TFA) and scavengers and after precipitated in ice-cold ethylic ether. The peptide was dissolved in water and freeze-dried. The crude peptide characterization was performed by electrospray ionization (ESI) LC-MS adopting a linear gradient of acetonitrile (0.1% TFA) in water (0.1% TFA) from 20 to 80% in 15 min. It was then purified by preparative reversed-phase high-performance liquid chromatography (RP-HPLC).

### Synthesis of *N*-Hydroxysuccinimide Ester of Folic Acid (FA-NHS)

FA-NHS was prepared by the following published method [28]. Five hundred milligrams of folic acid (FA) were dissolved in 10 ml of dimethyl sulfoxide (DMSO) with 240 ml of triethylamine. NHS (260 mg) and *N*,*N*-dicyclohexylcarbodiimide (470 mg) were added, and the mixture was reacted overnight at room temperature in the dark. The by-product, dicyclohexylurea, was removed by filtration. The DMSO solution was then concentrated under reduced pressure, and FA-NHS was precipitated in diethyl ether. The product was washed several times with anhydrous ether and dried in air.

### Synthesis of Functionalized MNPs

#### Synthesis of NH_2_@MNPs

MNPs (200 mg) were dispersed in H_2_O (25 ml) using an ultrasonic bath for 30 min. NH_2_-PA (100 mg) was added, and the suspension was agitated for 2 h at room temperature. The particles were separated magnetically and washed four times with H_2_O and then with ethanol and dried under air.

#### Synthesis of gH625@MNPs

NH_2_@MNPs (300 mg) and gH625 (1.2 mg) were dispersed in DMSO (20 ml). The solution was mixed overnight at 25 °C. The obtained particles were separated magnetically; washed with DMSO, H_2_O, and ethanol; and dried under air.

#### Synthesis of PEG,FA@MNPs

NH_2_@MNPs (300 mg), PEG-NHS (30 mg), FA-NHS (3 mg), and Rhod-NHS (3 mg) were dispersed in DMSO (15 ml), and the solution was mixed overnight at 25 °C. The obtained particles were separated magnetically; washed with DMSO, H_2_O, and ethanol; and dried under air.

### Sample Characterizations

X-ray powder diffraction (XRD) measurements were performed with a *θ*–*θ* 5005 Bruker-AXS diffractometer (Zeiss, Oberkochen, Germany) using Cu Kα radiation operating at 40 kV and 30 mA. X-ray photoelectron spectroscopy (XPS) was performed with a PHI 5600 multi-technique ESCA-Auger spectrometer with a standard Al-Kα X-ray source. Analyses were carried out with a photoelectron angle of 45° (relative to the sample surface) with an acceptance angle of ± 7°. The XPS binding energy (B.E.) scale was calibrated by centering the C 1s peak due to hydrocarbon moieties and adventitious carbon at 285.0 eV. UV/Vis measurements were carried out with a JASCO V-560 UV/Vis spectrophotometer, and the spectra were recorded with a ± 0.2 nm resolution. Dynamic light scattering (DLS) and zeta-potential measurements of MNPs were performed at 25 °C with a Zetasizer Nano-ZS (Malvern Instruments, Malvern, UK) equipped with He–Ne laser (633 nm) and a backscatter detector (173°). Transmission FT-IR measurements were recorded with a JASCO FTIR 430 spectrometer, using the KBr pellet technique, with 100 scans collected per spectrum (scan range 560–4000 cm^− 1^, resolution 4 cm^− 1^).

### Cell Culture

Human brain microvascular endothelial cells (HBMEC) and human glioblastoma cell line A-172 were grown to confluence in rat-tail collagen type I-coated tissue culture flasks, according to the reported method [29]. Briefly, HBMEC were grown in the endothelial cell medium supplemented with 5% fetal bovine serum (FBS) and 1% endothelial cell growth supplement. A-172 cell line was grown in DMEM containing 2 mM glutamine and 10% FBS as previously described [30]. In both cases, 100 U/ml penicillin and 100 μg/ml streptomycin were also added to the cultures.

### Cell Viability Assay

Cell viability was determined with the [3-(4,5-dimethyl-2-thiazolyl)-2,5-diphenyl-2*H*-tetrazolium bromide] (MTT) test [31]. In all experimental conditions, the FBS concentration was reduced to 1% (starving medium). Cells were seeded in 96-well plates at 7000 cells/well to obtain optimal cell density throughout the experiment. In all assays, cells were first incubated at 37 °C with MTT for 3 h; then, isopropanol with 0.04 M HCl was added, and the absorbance was measured at 1 h in a plate reader (Synergy 2-BioTek) with a test wavelength of 570 nm [32].

### Confocal Microscopy

Confocal microscopy was performed on both HBMEC and A-172 glioblastoma cells grown on microscope cover glasses placed in a 24-well plate. After incubations with or without MNPs, cells were fixed by adding 4% paraformaldehyde in PBS and processed for immunocytochemistry as early described [33]. Confocal microscopy analyses were carried out with an Olympus FV1000 confocal laser scanning microscope (LSM) equipped with the following excitation sources: diode laser (405 nm), multiline Ar laser (457, 488, and 515 nm), HeNe(G) laser (543 nm), and HeNe(R) laser (633 nm). An oil immersion objective (60xO PLAPO) was used, and the emitted light was detected in sequential mode through a spectral filtering system. The detector gain was fixed at a constant value, and images were taken for all of the samples at random locations throughout the area of the well. The following acquisition parameters were used: *λ*ex/em = 405/425 − 475 nm (blue channel, nuclear staining by DAPI); *λ*ex/em = 488/500 − 530 nm (green channel, gH625@MNPs labelling by NBD); and *λ*ex/em = 633/650 − 700 nm (red channel, PEG,FA@MNPs labelling by rhod). Quantitative analysis of fluorescence was performed by using the ImageJ software (1.50i version, NIH).

## Results and Discussion

### Synthesis and Characterization of MNPs

**Fig. 1:**
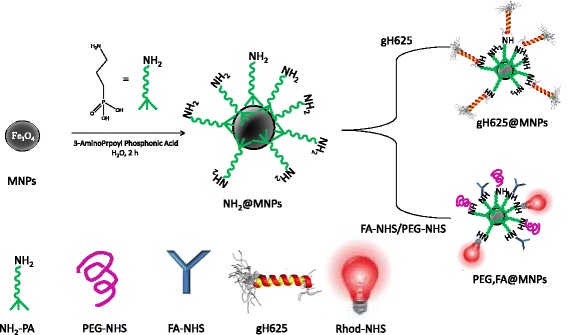
Reaction scheme. Reaction steps for the preparation of functionalized MNPs

The overall strategy implemented to prepare gH625@MNPs and PEG,FA@MNPs from Fe_3_O_4_ nanoparticles, obtained by co-precipitation, involves two steps as illustrated in Fig. 1. The first step, which is the same for both classes of nanoparticles, is based on the MNP functionalization with a phosphonic acid bearing an amino group (NH_2_-PA). In the second step, the NH_2_-PA-prefunctionalized MNPs (NH_2_@MNPs) are further conjugated with specific functional molecules via NHS coupling reactions. In particular, the *N*-hydroxysuccinimide-activated form of gH625 labeled with NBD was used for the preparation of gH625@MNPs, and *N*-hydroxysuccinimide activated forms of PEG, FA, and Rhod were used to obtain PEG,FA@MNPs. Note that both gH625@MNPs and PEG,FA@MNPs have been labeled with luminescent probes (i.e., NBD and Rhod, respectively) in order to carry out confocal laser scanning microscopy studies.

**Fig. 2:**
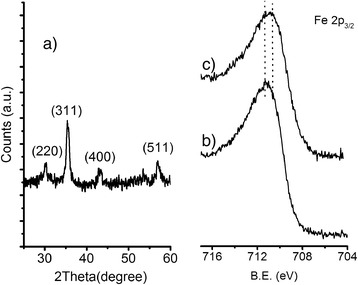
Typical XRD and XPS spectra of MNPs. XRD pattern of a) bare MNPs and high-resolution Fe 2p_3/2_ XPS spectral regions of b) bare MNPs and c) NH_2_@MNPs

The X-ray diffractometric (XRD) characterization of bare MNPs obtained after the co-precipitation step is similar to that reported in our previous papers [34, 35]. Briefly, a typical pattern (Fig. 2a) shows four diffraction peaks (2*θ* = 30.16°, 35.48°, 43.10°, and 57.04°) which is consistent with the presence of magnetite, maghemite, or any intermediate composition between the two phases. The lattice constant, *a*, that was found to be 8.389(4) Å in good agreement with the lattice parameter of bulk magnetite (8.396 Å), as well as the average crystal size of 11.2, determined applying the Debye–Scherrer’s equation, are comparable with the values previously reported [34, 35].

Note that since the crystal structure of Fe_3_O_4_ magnetite is very similar to that of γ-Fe_2_O_3_ maghemite, it is very difficult to distinguish the two phases by XRD. For this reason, X-ray photoelectron spectroscopy (XPS) was used to further analyze the MNPs. Figure 2b shows Fe 2p_3/2_ high-resolution XPS spectra of bare MNPs. The centroid of the band, observed at 710.9 eV, corresponds to the Fe 2p_3/2_ peak. This value is lower than that reported for Fe^3+^ either in octahedral holes of α-Fe_2_O_3_ (711.6 eV) [36, 37] or in mixed tetrahedral and octahedral holes of γ-Fe_2_O_3_ (711.4 eV), and it is consistent with the mixed oxidation state of Fe in Fe_3_O_4_ [37, 38].

Finally, a detailed magnetic characterization of similar MNPs is reported in our previous papers [34, 35].

The functionalization process was evaluated by FT-IR and XPS. After functionalization with phosphonic monolayers, the Fe 2p_3/2_ band (Fig. 2c) does not show relevant modifications, thus confirming that the Fe_3_O_4_ phase is retained. However, a slight shift (0.4 eV) of the Fe 2p_3/2_ centroid towards lower binding energy (B.E) was observed, which was associated to the anchoring of the phosphonic monolayer. Similar shifts were, in fact, observed in phosphate adsorption of various ferric oxides [39] and can be ascribed to some charge transfer from adsorbate to Fe atoms. The Fe 2p_3/2_ position of 710.5 eV was maintained after all the functionalization steps for the  anchoring of PEG and FA.

**Fig. 3:**
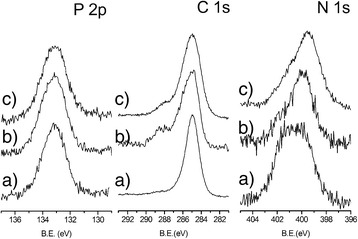
XPS characterization of functionalized MNPs. High-resolution P 2p, C 1s, and N 1s XPS spectral regions of a) NH_2_@MNPs, b) PEG,FA@MNPs, and c) gH625@MNPs

Figure 3 reports the comparison of P2p, N1s, and C 1s spectral regions of NH_2_@MNPs, PEG,FA@MNPs, and gH625@MNPs, respectively. The P2p peak position (133.2 eV) of NH_2_@MNPs is typically associated with the presence of the phosphonic acid anchored on the surface in a bidentate way [40–43]. The P2p band position in the spectra of gH625@MNPs and PEG,FA@MNPs is the same of that observed in NH_2_@MNPs spectrum, thus revealing that the phosphonic molecules are not removed during the coupling reactions for the peptide and folic acid immobilization.

The shape and the peak position of N1s spectra of NH_2_@MNPs are consistent with the presence of the anchored NH_2_-PA. The band consists of two distinct components: the first one at 399.9 eV is associated with the –NH groups of the anchored aminopropylphosphate, and the second component centered at 401.5 eV is due to the amino groups interacting with the surface of Fe_3_O_4_ through protonation or formation of –H bonds.

After the anchoring of either gH625 peptide or FA, PEG, and Rhod, the N1s component at 399.8 eV increases compared with the component at 401.8 eV related to protonated amino moieties of NH_2_@MNPs. This increment is due to the overlapped signals of the N atoms involved in the amidic bond (400.2 eV) between the anchored aminopropylphosphate and the conjugated molecules (e.g., gh625, FA, PEG, and Rhod) as well as of the N atoms of gh625 and FA (at 399.1 and 400.6 eV).

The C 1s band of NH_2_@MNPs spectrum consists of a single peak at 285.0 eV assigned to aliphatic carbons, as previously reported [40].

In the C1s spectra of gH625@MNPs and PEG,FA@MNPs, the presence of a C 1s component at 288.3 eV is due to the carboxylic and amidic groups of gh625, FA, and Rhod molecules.

**Fig. 4:**
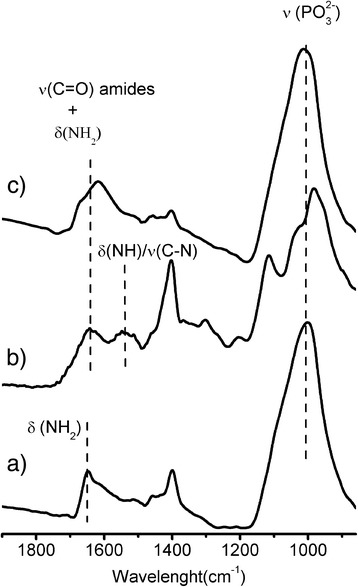
FT-IR characterization of functionalized MNPs. FT-IR spectral region in the 850–1900 cm^−1^ range  of a) NH_2_@MNPs, b) gH625@MNPs, and c) PEG,FA@MNPs

FT-IR spectra of NH_2_@MNPs, gH625@MNPs, and PEG,FA@MNPs are reported in Fig. 4. In all samples, spectra do not show the bands at 1250 cm^− 1^ due to P=O and the sharp peaks in the 900–1050 cm^− 1^ ranges due to P–O–H stretching [44–46], but a broad and strong band at 1040 cm^− 1^, which is associated to the vibrations of the PO_3_^2−^ group bonded to the iron surface. This band indicates, according to XPS results, that the phosphonic acids are deprotonated and are anchored to the surface through P–O–Fe bondings as previously reported [34, 40, 41]. The IR spectral region between 1500 and 1700 cm^− 1^ shows the bands belonged to the amino and amide groups of gH625 and FA. In particular, spectrum of NH_2_@MNPs shows a sharp peak around 1650 cm^− 1^ associated to the NH_2_ bending [47]. After PEG, Rhod, and FA anchoring, 1700–1500 cm^− 1^ region of PEG,FA@MNPs shows broader bands due to the convolution of the vibrations of unreacted NH_2_, amide groups, and benzene rings of FA and Rhod (1630–1600 cm^− 1^) [35]. Analogously, after peptide anchoring, the broad bands at about 1650–1600 cm^− 1^ can be due to the contribution of several vibrations due to unreacted NH_2_ and aminic moieties of the peptide side chains and to the C=O stretch of the peptide amidic bonds [48]. In addition, a component at about 1540 cm^− 1^ due to the combined *δ*(N–H)/ν(C–N) vibrations of the peptide [48] is also present.

**Fig. 5:**
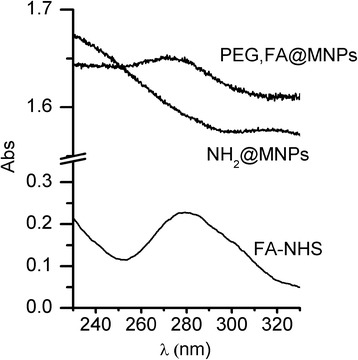
UV/Vis characterization of functionalized MNPs. UV/Vis spectra of a 5 μM FA-NHS solution and of NH_2_@MNPs, and PEG,FA@MNPs colloidal dispersions. Note that the high background observed in the spectra of NH_2_@MNPs and PEG,FA@MNPs is due to the scattering of the nanoparticle colloidal dispersion

The FA anchoring on MNPs has also been proved by the UV/Vis spectra of a PEG,FA@MNPs colloidal solution. Figure 5 compares the spectra of NH_2_@MNPs and PEG,FA@MNPs colloidal dispersions. The spectrum of a FA solution (5 μM) has been added as a reference. An evident band at 274 nm typical of the FA is clearly visible in both the reference and the PEG,FA@MNPs colloidal solution, thus confirming the presence of FA in the MNPs. Note that the slight shift is from the FA-NHS absorption at 280 nm to the value of 274 nm after anchoring is likely due to the change of the environment from free FA-NHS and nanoparticle-anchored FA. The presence of variable shifts of the absorption band at 280 nm after FA surface anchoring or conjugation with other molecules has been already observed in literature [49–51].

The mean hydrodynamic diameter, the polydispersity index (PDI), and the zeta potential of functionalized MNPs were determined by DLS in PBS buffer at pH 7.4 on MNP dispersions as prepared and after 72 h of aging (Table 1). The hydrodynamic diameters of NH_2_-PA@MNPs, gH625@MNPs, and PEG,FA@MNPs were 73.0 ± 3.0 nm, 104.0 ± 4.0 nm, and 51 ± 2 nm, respectively, and PDI values indicate a narrow enough distribution of the particle size. As expected, the conjugation with gH625 increased the size of nanoparticles, while the decrement in the size of PEG,FA@MNPs is due to a better dispersion than the NH_2_@MNPs, related to the presence of the PEG chains.

**Table 1:** Mean hydrodynamic diameter, polydispersity index (PDI), and zeta potential of functionalized MNPs determined by DLS in PBS as prepared and after 72 h of aging

	NH_2_@MNPs		gH625@MNPs		PEG,FA@MNPs	
	0 h	72 h	0 h	72 h	0 h	72 h
Size (nm)	73 ± 3	71 ± 4	104 ± 4	109 ± 4	51 ± 2	52 ± 6
PDI	0.23	0.22	0.18	0.20	0.34	0.37
*ζ* (mV)	− 44 ± 1	− 46 ± 1	− 45 ± 1	− 45 ± 1	− 42 ± 1	− 42 ± 1

In any case, note that highly negative zeta potential (< − 30 mV) were observed for all systems. Such negative zeta potential values ensure long-term stability and avoid extensive particle aggregation [52, 53]. Indeed, the negative surface charge was only slightly dependent on the coating but mainly resulted from the combination of the negatively charged core of Fe_3_O_4_ nanoparticles [54] and the effect of phosphonic acid groups as observed for similar systems [52, 34]. Therefore, compared to other synthetic strategies used to functionalize MNPs, the use of phosphonic acid monolayers as linkers not only allows the formation of stable bonding between the surface and the functional groups, but also induces highly negative zeta potential. After 72 h of aging, the size and zeta potential of NH_2_-PA@MNPs, gH625@MNPs, and PEG,FA@MNPs remain approximatively the same, suggesting that all decorated surfaces are stable over time.

### Cell Viability

**Fig. 6:**
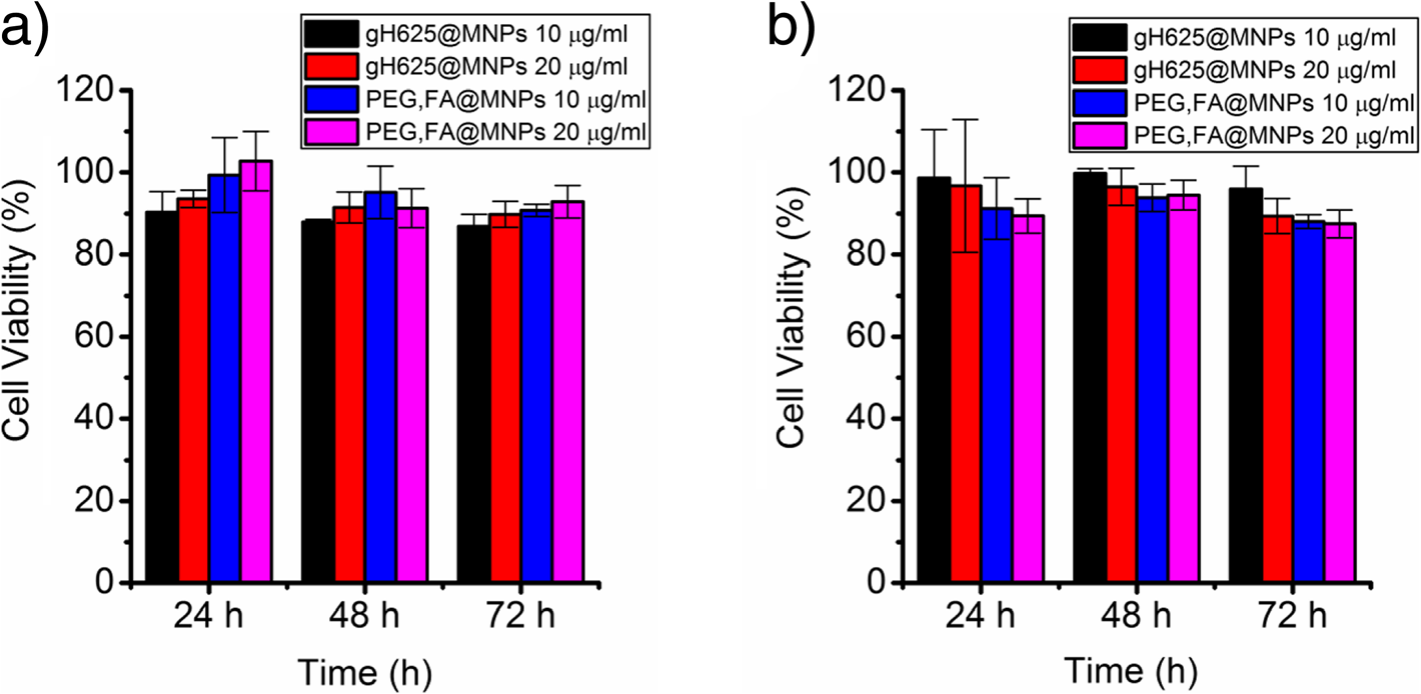
Cell viability. Cell viability of **a** HBMEC and **b** A-172 cells incubated for 24, 48, and 72 h with gH625@MNPs 10 μg/ml (black bar), gH625@MNPs 20 μg/ml (red bar), PEG,FA@MNPs 10 μg/ml (blue bar), and PEG,FA@MNPs 20 μg/ml (magenta bar)

The viability of HBMEC and A-172 cells incubated with gH625@MNPs and FA,PEG@MNPs was evaluated using MTT assay at different incubation times and in the presence of different concentrations of nanoparticles (10 and 20 μg/ml). As shown in Fig. 6, no concentration and/or time-dependent (24–48–72 h) cytotoxic effect of the decorated systems on HBMEC and A-172 cells was observed.

### Intracellular Uptake

In order to determine whether the cell-penetrating capability of gH625 peptide is retained after its immobilization at the nanoparticle surface, and to compare the gH625 and FA capability of crossing the BBB, the internalization into human brain endothelial cells of NBD-labeled gH625@MNPs and Rhod-labeled PEG,FA@MNPs has been investigated with confocal laser scanning microscopy.

**Fig. 7:**
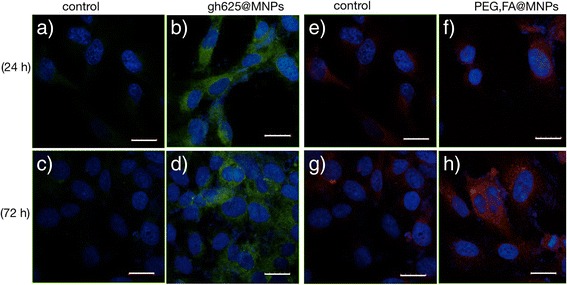
LSM fluorescence micrographs of HBMEC. LSM fluorescence micrographs of HBMEC incubated for 24 h (**b**, **f**) and 72 h (**d**, **h**) with NBD-labelled gH625@MNPs 15 μg/ml (**b**, **d**) and their controls (**a**, **c**) or rhod-labeled PEG,FA@MNPs 15 μg/ml (**f**, **h**) and their controls (**e**, **g**). Scale bar = 20 μm

**Fig. 8:**
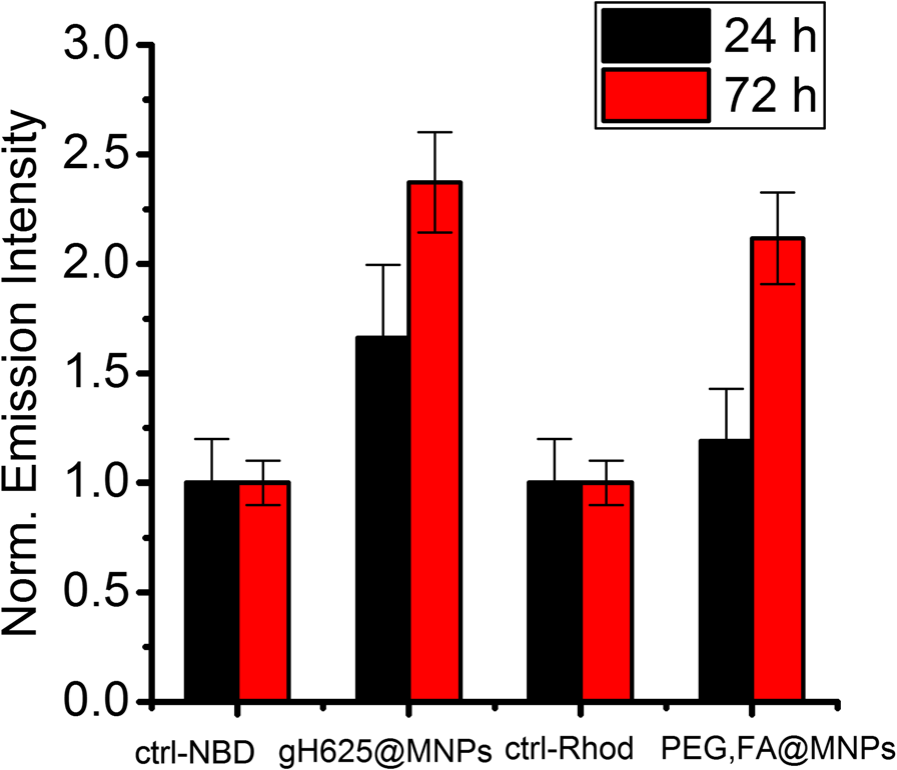
Normalized intensities of intracellular fluorescence. Quantitative analysis of intracellular fluorescence after HBMEC incubation at 24 and 72 h with NBD-labeled gH625@MNPs and Rhod-labeled FA,PEG@MNPs

After 24 h of incubation, the uptake of PEG-FA@MNPs is not evident, while gH625@MNPs internalization is clearly visible (Fig. 7). The conjugation with gH625 leads to a more rapid uptake and a nearly twofold higher intensity of the intracellular fluorescence (Fig. 8).

For longer times of incubation (72 h), differences between the internalization of the two systems are reduced. This behavior is not unexpected since for long incubation times, unspecific internalization processes of FA,PEG@MNPs can occur and our results further confirm what we previously reported for the uptake of functionalized and unfunctionalized polystirene nanoparticles [55].

To further explore the possibility of using gH625@MNPs as a therapeutic agent for brain tumors, the cellular uptakes of gH625@MNPs and FA,PEG@MNPs were investigated using the A-172 glioblastoma cell line. Glioblastoma is one of the most common primary brain tumor and also one of the most lethal cancers.

**Fig. 9:**
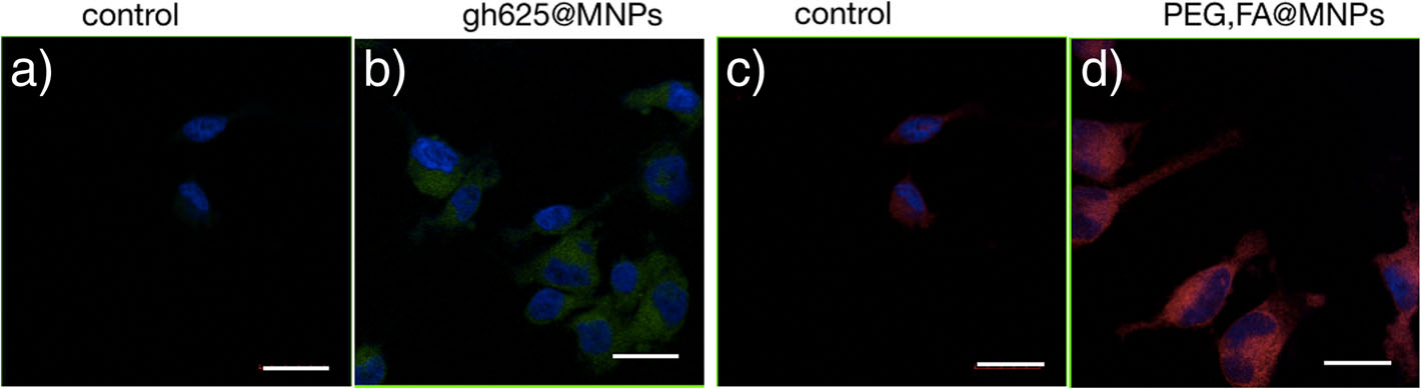
LSM fluorescence micrographs of glioblastoma cells. LSM fluorescence micrographs of glioblastoma cells. Merged blue-green images: control cells (**a**) and cells incubated with 15 μg/ml of NBD-labeled gH625@MNPs (**b**). Merged blue-red images: control cells (**c**) and cells incubated with 15 μg/ml rhod-labeled PEG,FA@MNPs (**d**). Scale bar = 20 μm

The cellular uptakes of gH625@MNPs and FA,PEG@MNPs after 24 h of incubation are comparable, as shown in Fig. 9.

This behavior suggests that the gH625 is capable of targeting brain tumor with the same efficiency of the more often adopted FA targeting unit.

## Conclusions

Fe_3_O_4_ nanoparticles have been functionalized with the gH625 viral cell penetrating peptide adopting a versatile route based on MNP prefunctionalization with a monolayer consisting of a bifunctional phosphonic linker, 3-aminopropylphosphonic acid. The cell internalization capabilities of this system have been evaluated by comparing them with those of a reference system based on MNPs functionalized with PEG, rhodamine, and folic acid, obtained adopting the same NH_2_-PA-based platform. The uptake of the two differently decorated MNPs was assessed in primary microvascular endothelial cells from human brain, which are the main components of the BBB and simulate an in vitro model of the BBB. These surface modifications influence the internalization of MNPs in HBMEC and, therefore, their capability to cross the BBB. In fact, conjugation with the gH625 peptide upgraded the delivery of nanoparticles across the in vitro BBB, leading to significant higher cell uptake in HBMEC after 24 h compared with that of FA bearing MNPs (FA,PEG@MNPs). Note that also other strategies have been used to enhance nanoparticle uptake across the BBB. A common approach is to attach targeting ligands in order to activate receptor-mediated endocytosis. As examples, transferrin-coupling nanoparticles can penetrate into the BBB through a transferrin receptor-mediated process [56]. Analogously, the linkage of the apolipoprotein E to the nanoparticles enhances the BBB penetration [57]. Besides, nanoparticles having a surface charges modified with polyethylenimine (PEI) has been reported to cross the BBB by absorptive-mediated transcytosis [58]. Finally, the ability of MNPs to pass through human brain microvascular endothelial cells, used as an in vitro BBB model, can be also facilitated by an external magnet [59]. In our work, we studied a different approach which use a cell penetrating peptide, the gh625, to improve internalization capabilities of Fe_3_O_4_ nanoparticles. These results are in accordance with those previously obtained by D. Guarnieri et al. using different kinds of MNPs [55, 60] confirming that the gH625-decorated magnetic nanoparticle has a relevant role in crossing the BBB and could be used as a safe and effective drug delivery system.

## Abbreviations

A-172: Human glioblastoma cell line
B.E.: Binding energy
BBB: Blood-brain barrier
CNS: Central nervous system
CPPs: Cell-penetrating peptides
DIPEA: *N*,*N*-Diisopropylethylamine
DLS: Dynamic light scattering
DMEM: Dulbecco’s modified Eagle’s medium
DAPI: 4′,6-Diamidino-2-phenylindole
DMSO: Dimethyl sulfoxide
FA: Folic acid
FA-NHS: Activated form of FA with NHS
FBS: Fetal bovine serum
Fmoc: 9-Fluorenylmethoxycarbonyl
FT-IR: Fourier transform infrared spectroscopy
gh625: 20-Residue peptide derived from the glycoprotein H (gH) of the Herpes simplex virus 1
gH625@MNPs: NH_2_@MNPs functionalized with gh625
HBMECs: Microvascular endothelial cells from human brain
MNPs: Magnetic iron nanoparticles
MTT: 3-(4,5-Dimethyl-2-thiazolyl)-2,5-diphenyl-2*H*-tetrazolium bromide
NBD: 4-Chloro-7-nitrobenz-2-oxa-1,3-diazole
NH_2_@MNPs: MNPs modified with NH_2_-PA
NH_2_-PA: 3-Aminopropylphosphonic acid
NHS: *N*-Hydroxysuccinimide
PBS: Phosphate-buffered saline
PDI: Polydispersity index
PEG: Polyethylene glycol
PEG,FA@MNPs: NH_2_@MNPs functionalized with FA, Rhod and PEG
PEG-NHS: Methoxypolyethylene glycol acetic acid *N*-succinimidyl ester
PEI: Polyethylenimine
Rhod: Carboxy-*X*-rhodamine
Rhod-NHS: Carboxy-*X*-rhodamine *N*-succinimidyl ester
TFA: Trifluoroacetic acid
XPS: X-ray photoelectron spectroscopy
XRD: X-ray powder diffraction
